# How do children’s cohabitation and family asset reserves impact rural individuals’ participation in commercial pension insurance?

**DOI:** 10.3389/fpubh.2025.1548241

**Published:** 2025-09-24

**Authors:** Chao Feng, Ying Zhang, Lulu Yang, Linjie Wu, Bin Liu

**Affiliations:** ^1^School of Economics and Management, Jiangxi Agricultural University, Nanchang, China; ^2^School of Economics and Management, Henan Agricultural University, Zhengzhou, China; ^3^Bureau of Agriculture and Rural Affairs, Ganzhou, China; ^4^Jiangxi Rural Development Research Center, Jiangxi Agricultural University, Nanchang, China

**Keywords:** participation behavior, family asset reserve, commercial pension insurance, children’s cohabitation, aging

## Abstract

This paper utilizes the Chinese Family Panel Studies (CFPS) database and employs the Probit model to examine the influence of cohabitation with children and household asset reserves on the engagement of rural people in commercial pension insurance. The study reveals that cohabiting with children markedly restricts rural residents’ engagement in commercial pension insurance. However, household asset reserves substantially enhance the probability of such participation. The significance and its direction remain unchanged following robustness and endogeneity assessments. The moderating effect indicates that the interaction term between household asset reserves and child cohabitation substantially increases commercial pension insurance participation, suggesting that household asset reserves mitigate the disincentive effect of child cohabitation. Heterogeneity analysis revealed that children’s cohabitation had a negative influence on participation in commercial pension insurance among residents with low educational attainment and poor health. In contrast, family asset reserves had a positive impact on participation in commercial pension insurance for residents with high educational attainment and good health. Consequently, we propose pertinent recommendations to enhance awareness of aging and transform perceptions of old age, augment farmers’ incomes and reduce insurance costs, assist prospective users, and refine insurance products.

## 1 Introduction

The aging challenge has garnered attention from countries worldwide due to a combination of factors, including the global increase in life expectancy (1, 2) and the ongoing decline in the fertility rate (3). The burden of old-age pensions exerts systemic pressure on sustainable fiscal development. It constrains strategic investments in scientific and technological innovation, education, and healthcare. It also results in an imbalance in the allocation of intergenerational resources, intensifying social structural tensions and instability. Diverse nations provide distinct solutions to the issue of aging. Japan, the most rapidly aging nation globally, is endeavoring to alleviate the challenges of aging by amending retirement legislation and fostering the silver economy. Europe, the most aged continent globally, employs cross-border pension arrangements to alleviate pension burdens. The United States, the most developed country in the world, uses dynamic regulation of skilled immigration quotas to cope with the old age dilemma.

**Figure 1:**
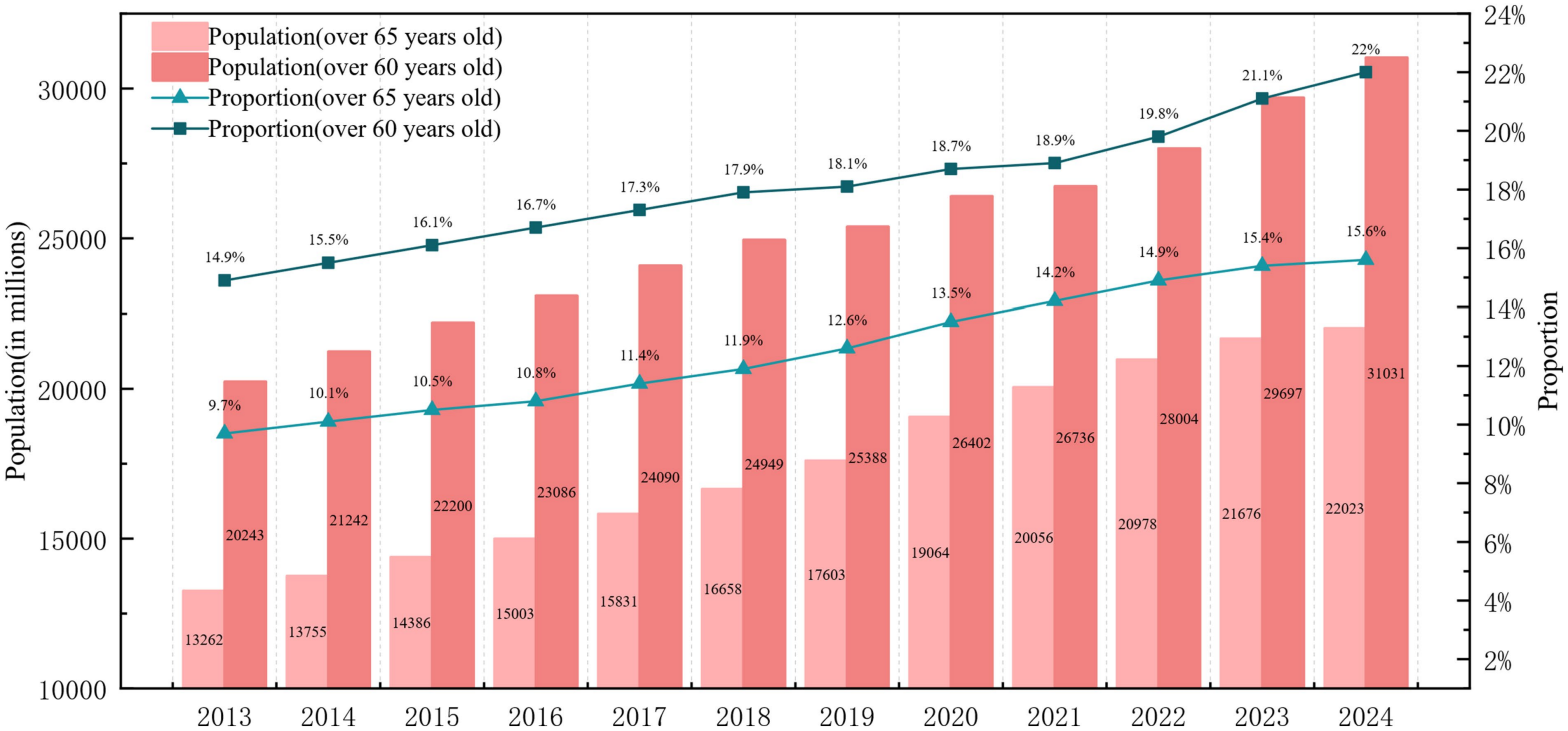
The trend of the older adult population in China from 2013 to 2024.

China’s older adult population base is vast, and the birth rate of China’s population has fallen sharply in recent years, especially in 2023, when China’s newborn population will be only 9.02 million, falling below the lowest value in history since 1949. This tendency has expedited the progression of China’s aging society. According to China’s seventh population census (2020), the population aged 60 years and over is 264.02 million, comprising around 120 million individuals in rural areas. However, by the end of 2024, China’s older adult population aged 60 and above had reached 310.31 million, accounting for 22.0% of the total population, and the population aged 65 and above had reached 220.23 million, representing 15.6% of the total population (Figure 1). This rate of aging exacerbates China’s future burden of old age. Consequently, the Chinese government has urged the expedited development of the pension insurance system to mitigate the challenges posed by the growing older population (4, 5). The risks of imbalance and payment deficits in basic pension insurance (the first pillar), along with the limitations of restricted coverage and fewer investment avenues in corporate and occupational pensions (the second pillar), have resulted in a potential fiscal burden of pensions in China (6, 7). Simultaneously, Chinese citizens, particularly in rural regions, are constrained by traditional cultural perspectives and a deficiency in pension planning awareness, exacerbating the issue of aging in rural China. Consequently, the Chinese government must investigate innovative solutions for aging to alleviate the societal issues stemming from the excessive weight of an aging population.

The Chinese government has emphasized the advancement of the third pillar of pensions, represented by commercial pension insurance, which is crucial in alleviating the strain on the first and second pillars and addressing the challenges of rural aging. Implementing advantageous policies (8), including the tax-deferred pension insurance pilot program and the pension insurance preferential policy for rural regions, has markedly enhanced the density and depth of participation in commercial pension insurance. Additionally, the participation density was RMB 136 per person, representing a 29.1% year-on-year increase. Rural commercial pension insurance has alleviated the burden of intergenerational family assistance, facilitated the transfer of pension assets across different periods, and enhanced the structure of pension risk management, thereby substantially contributing to resolving the pension crisis in rural regions. However, the aging environment in China’s rural areas is complex. On the one hand, the “children living together in the same household” is still the most important old-age pension program in rural China. Influenced by Confucianism and the traditional culture of filial piety, the proportion of rural Chinese residents whose fathers continue to live with their children in old age remains high. Although the percentage of three-generation households in rural China has decreased, 78.6% of rural older adult individuals continue to reside together as their primary mode of housing, as per the China Rural Development Report (2023) (9). In addition, given the sudden acceleration of China’s aging society, which has resulted in a situation of “old age before preparedness,” children’s co-parenting remains the primary resource for coping with old-age difficulties in rural areas. Therefore, the “children’s cohabitation” model will have a crowding-out effect on commercial pension insurance. On the other hand, household asset reserves are reshaping rural residents’ retirement decisions. The median household financial assets of rural residents in China will increase from 21,000 yuan in 2015 to 68,000 yuan in 2022 as a result of China’s comprehensive poverty eradication policy, inclusive financial policy, and the policy of transferring collectively operated construction land to the market. Through mechanisms such as enhanced risk buffers, strengthened intergenerational substitution, and increased ability to pay, increasing household asset reserves establish an economic foundation for rational and diversified decision-making in the context of old age. As a result, the increase in household asset reserves has also led rural residents to favor commercial pension insurance when faced with retirement choices.

In this complex context, it is imperative to explore the profound logic of the actual pension status of Chinese rural residents on pension decision-making and to explore the impact of children living together and family asset reserves on rural pension insurance participation decision-making. Firstly, it plays an important role in understanding the current situation and the old-age tendency of rural residents. Secondly, This holds significant reference and directional value for increasing participation in rural business pension insurance, thus facilitating the advancement of China’s pension system and mitigating the burden of rural pensions. Thirdly, Chinese-style pension plans should be contributed to other countries to alleviate the world’s pension problems.

## 2 Examination of current research literature

### 2.1 Research on cohabitating children and commercial pension insurance

There are few studies about the influence of children residing at home on engagement in commercial pension insurance; however, two contrasting perspectives have surfaced. One perspective posits that cohabitation with children enhances engagement in pension insurance. From the standpoint of information spillover, commercial pension insurance, as a distinctive financial instrument that enhances wealth and mitigates risks, significantly influences the decision to acquire pension insurance, with financial literacy and risk attitudes being pivotal factors (10, 11). In the presence of cohabiting children, regular communication can enhance financial literacy, refine asset allocation, and mitigate family financial vulnerability (12). It also significantly improves the likelihood of citizens engaging in business insurance (10). From the standpoint of intergenerational support, the cohabitation of children with their parents is attributable to intergenerational caring (13). In rural regions, the arrival of grandkids enhances the probability of “retirement” for older family members (14). This intergenerational activity can liberate labor (15), improve income levels, and engage in commercial pension insurance (16). Should this intergenerational engagement cease, the old will experience an increase in depressed symptoms, hence diminishing the quality of care (17). Another perspective suggests that cohabiting with children may harm engagement in commercial pension insurance. From a global perspective, intergenerational care is a prevalent form of familial labor in which older adult individuals participate (18). Parents may cohabit with their offspring due to intergenerational caregiving, and both intergenerational labor and financial assistance will progressively transition to grandchildren (19). Parents would allocate the resources and capital they would have directed toward the financial market in the intergenerational care relationship, diminishing their financial reserves and inhibiting rural residents’ engagement in commercial pension insurance. Conversely, the primary tasks of familial support in advanced age encompass financial assistance, caregiving, and spiritual solace (20). Children serve as a primary resource for rural inhabitants in their later years, as the financial assistance they offer mitigates the material burden of elder support, the care they render diminishes the behavioral demands of elder care, and their emotional companionship alleviates the psychological stress associated with the elder backing. Consequently, this reduces the necessity for preventive reserves for elder care and exerts a notable “substitution” effect on commercial elder insurance (21).

### 2.2 Research on asset reserves and business pension insurance

A substantial body of literature confirms that income level has a positive influence on pension savings and participation in commercial pension insurance (16, 22–24). Several experts have validated through surveys conducted in the Netherlands (25) and Australia (26) that residents’ income levels significantly influence pension savings. Middle- to high-income individuals are more likely to select commercial pension insurance (27). As income levels increase, they tend to engage in pension insurance programs with elevated contribution levels (28). Nonetheless, low-income individuals save minimally for retirement and often lack plans for their later years (29). Specific experts have discovered that income levels do not exhibit a linear correlation with individuals’ propensity to engage in commercial pension insurance; instead, the relationship resembles an inverted “U”-shaped curve (30, 31). Participation in commercial pension insurance is contingent upon a wealth threshold, leading high-income individuals to opt for riskier financial assets with more significant returns, diminishing their likelihood of engaging in commercial insurance. Pension insurance is the primary source of income for maintaining the quality of life in old age (32); however, commercial pension insurance features a prolonged investment cycle, leading residents to prioritize family asset reserves over temporary income increases (33). In contrast to short-term total monetary income, household asset reserves—representing the cumulative wealth owned by a household over an extended period—offer more stable financial support for managing financial activities, such as retirement planning. Households possessing more fabulous real estate and vehicles exhibit a higher propensity to engage in commercial pension insurance (34), indicating that asset reserves positively influence participation in such insurance (35).

### 2.3 Research on additional elements and business pension insurance

Furthermore, scholarly investigations into commercial pension insurance can be categorized into three dimensions. Initially, in terms of characteristics, factors such as residents’ educational attainment (36), risk propensity (37), and financial literacy (38, 39) positively influence enrolment in commercial pension insurance. Furthermore, families with fewer offspring can enhance the quality of life for the older adult (40), and the likelihood of a sole child acquiring pension insurance for their parents is increased (41). As the population of children rises, the marginal substitution rate between family and personal pensions diminishes (42). Secondly, it is from the standpoint of the concept of eldercare. In rural regions, conventional “family-oriented” older adult care substantially displaces commercial older adult insurance (43). Subsequently, due to social and cultural transformations, there has been a progressive transition toward a contemporary paradigm of elder care (44). This novel model has improved access to commercial old-age insurance and, through the interplay of traditional and modern ideas, has also enhanced aged-care services (45). Third, it is viewed from the standpoint of social interaction. Social mutual aid can be categorized into the effects of the partner and demonstration group (46). The peer group effect can influence membership in commercial pension insurance through three dimensions: disseminating verbal information (47), exchanging usage experiences with peers (48), and social normative mechanisms (49). The group demonstration effect influences the decision to obtain commercial pension insurance by disseminating usage experiences (50). A high perceived value encourages involvement in commercial insurance, whereas a low perceived value discourages it.

Current research provides significant theoretical support for this paper’s examination of rural citizens’ engagement in commercial pension insurance; however, deficiencies remain. Most extant literature primarily addresses the first and second pillars of pension insurance, with a notable lack of focus on business pension insurance. Secondly, most scholars have not sufficiently considered the complex context of children living together and increasing asset reserves and have concentrated solely on the impact of a single factor on commercial pension insurance. Lastly, most of the current literature collectively examines the experiences of rural and urban residents. However, fewer samples of rural residents are examined separately. Therefore, this study utilizes the China Family Panel Studies (CFPS) databases from 2018 and 2020 to examine the effects of children’s cohabitation and family asset reserves on rural residents’ engagement in commercial old-age insurance, employing empirical methodologies including the Probit model, marginal effects model, and moderating effects model. Specifically, the marginal contributions of this paper lie in the following three areas: First, we incorporate children’s cohabitation and household asset reserves into the same analytical framework, analyzing the distinct impacts of these factors on rural residents’ participation in commercial pension insurance within a complex context. Second, this study focuses on the old-age choice of rural residents to provide theoretical support for alleviating rural old-age problems. Third, This study verifies this relationship by examining the moderating influence of the interplay between children’s cohabitation and household asset reserves on commercial pension insurance.

## 3 Theoretical framework and research hypotheses

### 3.1 Examination of the correlation between cohabitation with offspring and engagement in commercial pension insurance

Mr. Fei Xiaotong’s “Feedback Theory” provides a theoretical foundation for comprehending children’s involvement in the household (51), highlighting their duty to reciprocate to their parents. Parents perceive that they nurtured their progeny throughout their youth. In return, their children provide support in their old age, constituting a tangible expression of gratitude for their upbringing. In rural regions, this feedback behavior is particularly significant, as the gradual migration of the young labor force to urban areas exacerbates the loneliness experienced by the old. Consequently, rural parents desire feedback that encompasses both practical care and spiritual comfort (52), which constitutes the fundamental role of the rural family. Moreover, intergenerational transfer theory is a significant theoretical foundation, highlighting the reciprocal exchange of resources among family members, particularly the transfer of economic resources from the younger generation to the older age. The scarcity of labor markets in rural regions and the inadequate economic independence of older adult parents enable children residing with them to provide direct support (53), thereby reducing the need for commercial pension insurance for the old. This assistance reduces the necessity for seniors to have private retirement insurance. Children residing with their parents align with familial obligations and cultural norms, as outlined in the “feedback theory,” as well as the concept of intrafamily resource allocation in the theory of intergenerational transfers. This dual process prompts rural dwellers to prefer reliance on familial support systems over external commercial insurance products in their retirement planning decisions.

Consequently, the subsequent hypotheses are posited:

*H1*: The presence of children in the same household may impede rural inhabitants’ engagement in commercial pension insurance.

### 3.2 Analysis of the correlation between household asset reserves and rural people’s engagement in commercial pension insurance

Income level and family asset reserves are the primary metrics used to assess a family’s economic standing. The income level represents the short-term cash intake of a household, indicating its daily consumption capacity and the fundamental quality of life. In contrast, a family asset reserve refers to the long-term accumulation of wealth. Commercial pension insurance, as a long-term and consistent investment in financial products, may be adversely affected by short-term income fluctuations, rendering rural residents unable to sustain long-term premium payments. Consequently, examining the importance of family assets in relation to participation in pension insurance is particularly significant. According to life cycle theory, individuals exhibit varying consumption and saving objectives at different stages of life (54). The labor income of parents during their youth is primarily allocated for child-rearing and family establishment. As they age, their labor income diminishes, and previously amassed assets become a crucial resource for maintaining a high quality of life in later years. To secure a steady financial resource in later life and alleviate the burden of caring for aging children, a portion of family assets will be allocated to acquire commercial pension insurance, ensuring future economic stability and facilitating a seamless transition through various life stages. According to asset allocation theory, after rural inhabitants accumulate sufficient family asset reserves, they will allocate a portion of their assets to commercial pension insurance to optimize asset distribution and mitigate risks. Commerce pension insurance facilitates the diverse allocation of assets. It mitigates the impact of a single asset’s risk on the family’s overall economic condition. Simultaneously, Commercial pension insurance provides a dependable source of pension funds, supplementing other assets and collectively safeguarding rural residents in their later years.

Consequently, the subsequent hypothesis is posited:

*H2*: Household asset reserves will enhance rural people’s engagement in commercial pension insurance.

## 4 Data sources, variable selection, and model selection

### 4.1 Data sources

The data used in this paper are derived from the 2018 and 2020 China Family Panel Studies (CFPS2018 and CFPS2020) databases, which employ a stratified sampling methodology during the survey phase and encompass 31 provinces (including autonomous regions and municipalities directly under central government jurisdiction) in China. The survey questionnaires are categorized into four primary themes: adults, children, families, and communities. They encompass extensive information regarding the individual’s current status, family economy, and social economy, thereby offering reliable data to understand the economic behavior of Chinese families. Given the low participation rates among rural residents in commercial pension insurance in China, relying solely on a single year’s data may yield inconclusive results. Consequently, the methodology employed in this paper is as follows: first, it integrates the databases from 2018 and 2020, subsequently eliminating duplicate samples based on individual codes while retaining the personal data from 2020; second, China’s prevailing retirement age is 55–60 years, and commercial pensions, as a continuous investment financial instrument, are acquired for a duration of 15–20 years. This paper eliminates samples over 40 years old and those under 40; additionally, it focuses on rural residents, filtering out data from those residing in “towns.” Ultimately, we exclude the missing data and evident abnormalities in the explanatory factors, core explanatory variables, and control variables, resulting in the retention of 1,583 valid samples.

### 4.2 Variable selection

#### 4.2.1 Dependent variable

The dependent variable in this study is “participation in commercial pension insurance,” a binary dummy variable indicated by the questionnaire item “Which types of pension insurance have you participated in?” If the answer options presented to the inhabitants contain “commercial pension insurance,” the value is designated as “1”; if the answer options do not include “commercial pension insurance,” the value is defined as “0.”

#### 4.2.2 Principal explanatory variable

Cohabiting children. This article defines”Cohabiting children” based on existing literature (55). This study employs the phrase “whether living at home” to delineate the real living arrangements of parents and children. Sons or daughters who depart from home for 3 months or less and subsequently return are considered to be residing with their parents. The value is designated as “1.” Sons or daughters who depart from home for a duration exceeding 3 months without returning are deemed to be residing independently from their parents. This circumstance is designated a value of “0.” Furthermore, the CFPS database contains a question that can illustrate the economic relationship between parents and their offspring. If the respondent does not need to support the current family financially, nor does the current family require financial support, they are deemed economically independent and assigned a value of “1”; otherwise, the value is “0.” This measure captures the economic interactions between parents and children but does not encompass other dimensions of intergenerational support in cohabitation; hence, this variable is utilized exclusively for endogeneity testing.

Residential asset reserves. Household asset reserves can indicate the wealth of the respondent’s family, representing not just purchasing power but also significantly influencing the residents’ selection of retirement model. Cash, real estate, and automobiles are the three prevalent forms of household asset reserves. This research identifies household asset reserves as the principal explanatory variable, with cash holdings, the number of real estate properties, and car ownership as supplementary explanatory factors. This paper utilizes the response to the query “What is the total amount of cash and deposits of all current family members in your household?” to quantify the total assets of resident households. It subsequently applies the logarithm of the total assets to denote the household’s cash holdings. In the initial phase, the responses to the inquiry “Who owns the house you are currently living in?” in the questionnaire are evaluated, with responses indicating “family members own all or part of the property rights” receiving a value of “1,” and all other responses receiving a value of “0.” In the second step, refine the responses to the question, “How many additional properties do you or other family members possess?” in the questionnaire. In the third step, aggregate the values derived from the preceding two phases to ascertain the total number of attributes. The response to the question indicates cars “Does your household own a car?” In the questionnaire, with a value of “1” for an affirmative answer and “0” for a negative response.

#### 4.2.3 Regulate variables

This research includes nine control variables, encompassing person characteristics, family characteristics, and risk attitudes, to comprehensively describe the factors influencing rural individuals’ involvement in commercial pension insurance. Personal attributes encompass age, gender, marital status, educational attainment, health condition, and “age squared/100” of rural inhabitants. Family characteristics pertain to the number of family members, while risk attitudes comprise the dimensions of risk appetite and risk assets. Concerning risk appetite, the variable is determined by the methodology of Zhao et al. (56), which assigns a value of “1” to the option where the interviewee chooses to flip a coin, receiving 200 yuan for heads and 0 yuan for tails, indicating a higher risk appetite among residents. Conversely, the option of forgoing the coin flip for a guaranteed 100 yuan is assigned a value of “0,” reflecting a lower risk appetite. When the respondent receives 100 yuan directly, the assigned value is “0,” indicating a diminished risk appetite (56). For risk assets, a value of “1” is assigned if the responder possesses stocks, funds, government bonds, trust products, or foreign exchange products; otherwise, a value of “0” is assigned. Tables 1, 2 present the definitions and assignments of each variable.

**Table 1:** Variable definitions and assignments.

Variable type	Variable symbol	Variable name	Variable definition and assignment
Dependent variable	Insure	Commercial pension insurance	Have you purchased commercial pension insurance? No = 0; yes = 1
Principal explanatory variables	Living	Children living together	Does one live in this household? Not living = 0, living = 1
Principal explanatory variables (family asset reserve)	Cash	Household cash reserves	The logarithm of total household assets (cash and deposits with financial institutions) of
	House	Number of houses	How many houses does the interviewee own?
	Car	Whether or not you own a car	Does the respondent’s household own a car? No = 0; yes = 1
Control variable	Age	Age	Age of interviewee (years old)
	Gender	Gender	Female = 0; Male = 1
	Marriage	Marriage	Other = 0; Married = 1
	Education	Years of schooling	Never attended school, illiterate/semi-literate = 1; Primary school = 6; Junior high school = 9; High school/technical school/vocational school = 12; College and above = 15; Bachelor’s degree = 16; Master’s degree = 19
	Health	Degree of health	Respondents’ health status: unhealthy = 1; average = 2; relatively healthy = 3; very healthy = 4; extremely healthy = 5
	Age^2^/100	Age^2^/100	Square of the age of the interviewee/100
	Size	Household size	Number of people in the respondent’s household
	Risk	Risk attitude	Dummy variable: respondents who chose “get $100 directly” = 0, and those who chose “toss a coin, heads you get $200, tails you get $0” = 1
	Finance	Whether or not they hold financial assets	According to the questionnaire “Do you currently hold any financial products such as stocks, funds, government bonds, trust products, or foreign exchange products?,” no = 0; yes = 1

**Table 2:** Descriptive statistics for each variable.

Variable	Obs	Mean	Std. dev.	Min	Max
Insurance	1,386	0.100	0.293	0	1
Living	1,386	0.321	0.467	0	1
Cash	1,386	7.443	4.413	0	15.320
House	1,386	1.120	0.533	0	8
Car	1,386	0.274	0.446	0	1
Gender	1,386	0.553	0.497	0	1
Marriage	1,386	0.906	0.292	0	1
Age	1,386	45.187	4.350	40	55
Age^2^/100	1,386	20.608	4.069	16	30.250
Health	1,386	2.972	1.240	1	5
Education	1,386	5.525	4.810	0	19
Size	1,386	4.557	1.854	1	21
Risk	1,386	0.242	0.428	0	1
Finance	1,386	0.020	0.141	0	1

**Figure 2:**
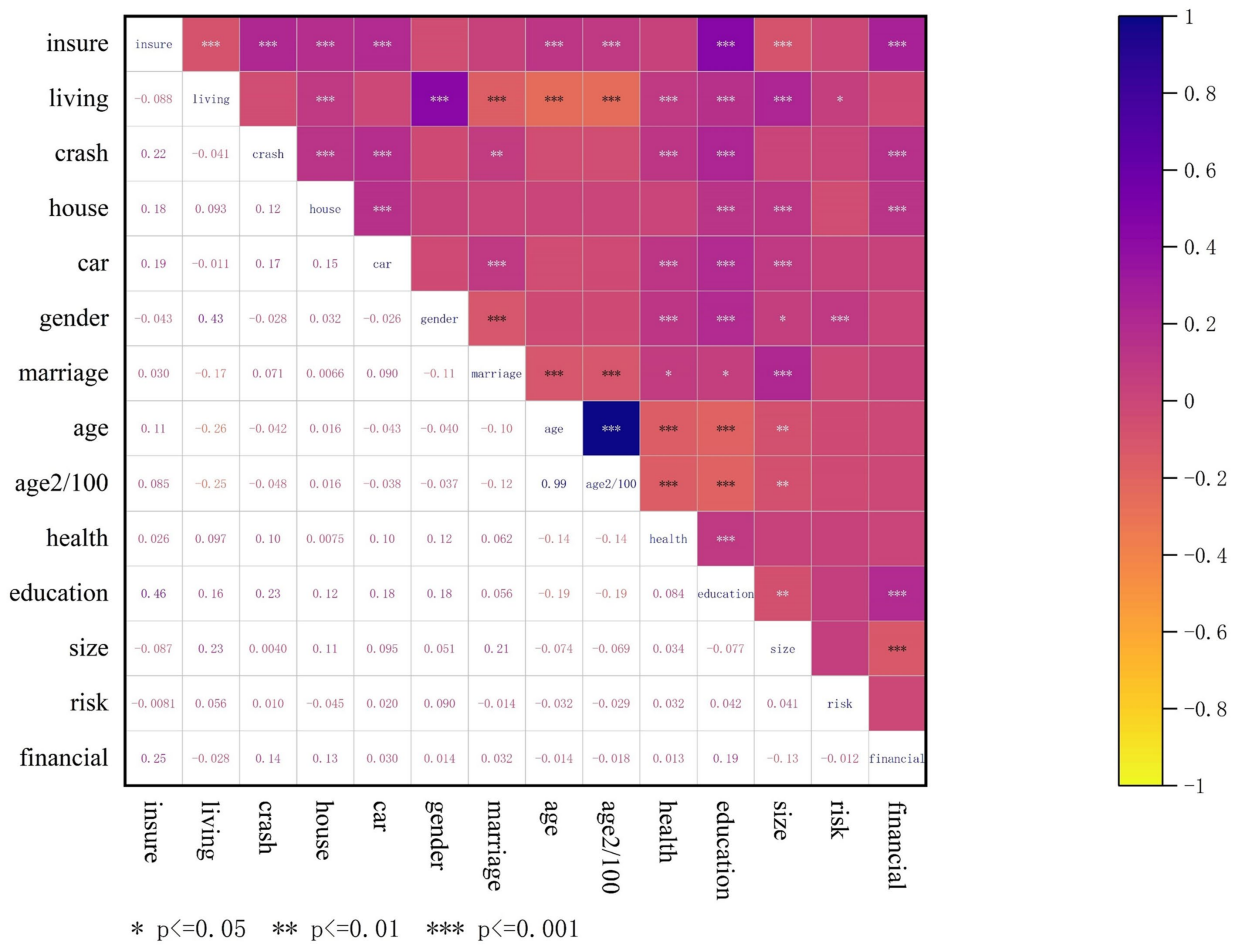
Correlation heat map of variables.

This study utilizes a coefficient heat map to analyze the correlation among the distinctive factors. The proximity of the small square’s color to purple indicates a stronger positive correlation between the two variables. In comparison, a closer resemblance to yellow signifies a stronger negative correlation. Suppose a characteristic variable exhibits a strong correlation with the output label yet shows little to no correlation with the output labels of other variables. In that case, it indicates that this variable is not a redundant feature. If two variables are perfectly correlated, they signify that they are redundant representations of one another. The correlation coefficient heat map in Figure 2 indicates that aside from the two feature variables, age and “age^2^/100,” the remaining selected variables exhibit weak correlations. Consequently, the feature variables in this study satisfy the fundamental criteria for regression analysis.

#### 4.2.4 Model selection

Probit model. The dependent variable in this study is “the decision to purchase commercial pension insurance,” which represents a standard binary classification variable. A Probit model is developed, incorporating the attributes of the CFPS database, to analyze the determinants influencing rural residents’ engagement in commercial pension insurance, including the presence of children at home and family asset reserves. The particular Probit model is delineated as follows:


(1) $$\text{Probit}({\text{insure}}_{i}=1)=\Phi (\begin{array}{l} \alpha +\beta_{1}{\text{living}}_{i}+\beta_{2}{\text{crash}}_{i}\\ +\beta_{3}{\text{house}}_{i}+\beta_{4}{\mathrm{car}}_{i}+{\text{γControl}}_{i}+\varepsilon_{i} \end{array})$$

In Equation 1, ${\text{insure}}_{i}\;$is the explained variable, which indicates the participation of rural residents in commercial pension insurance; ${\text{living}}_{i}\;$is the co-residence of children of rural residents;$\;{\text{crash}}_{i}$ is the cash holdings of rural residents;$\;{\text{house}}_{i}$ is the number of real estate of rural residents;${\mathrm{car}}_{i}\;$is the car ownership of rural residents; ${\text{Control}}_{i}$ is the control variable described above;α is the intercept parameter;$\beta_{1},\beta_{2},\beta_{3},\beta_{4},\gamma \;$is the slope parameter, and $\varepsilon_{i}$ is the residual term.

## 5 Empirical findings and analysis

### 5.1 Analysis of initial regression outcomes

Table 3 presents the Probit baseline regression outcomes. Model (1) solely incorporates the influence of children residing at home and household asset reserves on engagement in commercial pension insurance, whereas models (2) to (4) progressively introduce control variables with varying attributes. The Probit model can indicate the direction and importance of results but cannot quantify the degree of influence of each variable on commercial pension insurance. Consequently, this work presents the average marginal effect, with the outcome being model (5).

**Table 3:** Probit benchmark back.

	Model (1)	Model (2)	Model (3)	Model (4)	Model (5)
	Baseline	Baseline	Baseline	Baseline	Marginal effect
Living	−0.490^***^	−0.478^***^	−0.472^***^	−0.459^**^	−0.459^**^
	(0.127)	(0.172)	(0.180)	(0.181)	(0.181)
Cash	0.108^***^	0.042^**^	0.042^**^	0.037^**^	0.037^**^
	(0.017)	(0.018)	(0.018)	(0.018)	(0.018)
House	0.390^***^	0.324^***^	0.325^***^	0.311^***^	0.311^***^
	(0.086)	(0.096)	(0.097)	(0.097)	(0.097)
Car	0.531^***^	0.466^***^	0.467^***^	0.473^***^	0.473^***^
	(0.105)	(0.134)	(0.135)	(0.136)	(0.136)
Age		1.531^***^	1.527^***^	1.457^***^	1.457^***^
		(0.370)	(0.372)	(0.374)	(0.374)
Gender		−0.237^*^	−0.237^*^	−0.257^*^	−0.257^*^
		(0.142)	(0.142)	(0.144)	(0.144)
Marriage		−0.236	−0.227	−0.262	−0.262
		(0.263)	(0.272)	(0.269)	(0.269)
Education		0.193^***^	0.193^***^	0.189^***^	0.189^***^
		(0.016)	(0.016)	(0.016)	(0.016)
Health		0.048	0.048	0.051	0.051
		(0.057)	(0.057)	(0.057)	(0.057)
Age^2^/100		−1.509^***^	−1.504^***^	−1.429^***^	−1.429^***^
		(0.392)	(0.394)	(0.396)	(0.396)
Size			−0.005	0.007	0.007
			(0.039)	(0.040)	(0.040)
Risk				0.029	0.029
				(0.151)	(0.151)
Finance				0.725^**^	0.725^**^
				(0.333)	(0.333)
PseudoR^2^	0.1647	0.4684	0.4684	0.4740	–
*N*	1,386	1,386	1,386	1,386	1,386

Robust standard errors in parentheses ***, **, and * indicate significance at the 1, 5, and 10% levels, respectively.

Models (1) to (5) indicate that residing with children substantially hinders rural inhabitants’ engagement in commercial pension insurance. With each new child in the home, the likelihood of rural inhabitants engaging in commercial pension insurance diminishes by 4%. It initially corroborates Hypothesis H1.

According to Model (1) to Model (5), children living in the same household significantly inhibited rural residents’ participation in commercial pension insurance. Hypothesis H1 was preliminarily verified. From the children’s perspective, as the father’s physical capabilities and financial status deteriorate, the children offer intergenerational support to him, which they perceive as a duty that diminishes the probability of engaging in insurance. From the perspective of their parents’ generation, the new model of old-age care has not been accepted by their parents. When they face the old life, they not only yearn for the life care and spiritual comfort provided by their children but also have a deep fear of “no place for the old” in the new old-age care mode. The phenomenon is because fathers and mothers living with their children add a sense of well-being and security in old age, an emotional value commercial pension insurance cannot provide. From an economic standpoint, cohabitating children with the old necessitates increased pension expenditures for the family. To alleviate familial economic strain, family members and the older adult will prioritize family pensions in pension decisions, thereby diminishing the need for insurance. From the perspective of cultural attitudes, raising children for old age is still the primary mode of old-age care in rural China. It is challenging for rural residents to adapt to the changes introduced by the new mode of care for the older adult. Moreover, rural residents still generally uphold the concept of “raising children for old age,” support for the older adult is seen as an important manifestation of filial piety; family members may be more willing to take care of the older adult through practical actions. From a cultural standpoint, child-rearing by the older adult remains the predominant form of eldercare in rural China, and rural inhabitants are resistant to the alterations introduced by contemporary eldercare models. Moreover, rural inhabitants typically adhere to the principle of “raising children to support the older adult,” viewing assistance to seniors as a significant manifestation of “filial piety.” Consequently, family members may be more willing to care for the older adult through tangible acts of kindness. Consequently, children’s cohabitation has a clear crowding-out impact on rural residents’ engagement in commercial pension insurance.

Models (1) to (5) demonstrate that household asset reserves substantially enhance rural households’ engagement in commercial pension insurance. Moreover, each unit’s rise in cash holdings, property ownership, and car ownership correlates with an increased likelihood of engaging in commercial pension insurance, initially substantiating Hypothesis H2. In the context of reshaping the mechanism of risk appetite, The risk propensity is altered by the asset reserve, which increases the certainty of future income. The life cycle consumption curve is smoothed by commercial pension insurance, which converts current income into long-term security, allowing rural residents to enhance their risk tolerance through institutional security. It not only induces farmers to transition from “precautionary savings” to “preventive investment,” but it also alleviates their anxiety regarding potential fluctuations in cash flow. The asset reserve undermines the path dependence on children’s old-age care by establishing institutionalized security from the perspective of an intergenerational contract replacement mechanism. The family asset reserve is an economic substitute for the family’s internal support when converted into pension insurance rights and interests. This substitution effect is especially significant in intergenerational cohabitation families, as it disrupts the closed cycle of implicit contracts and transfers the responsibility of support from an ethical obligation to a market contract. From the standpoint of enhancing financial literacy mechanisms, asset accumulation is concurrent with the development of financial literacy. Farmers progressively acquire financial skills by engaging in contract signing, income calculation, risk assessment, and other operations, as asset accumulation is a lengthy process. Farmers are increasingly recognizing the numerous benefits of commercial pension insurance due to cognitive upgrading, changes in risk perception, and improved decision-making. Therefore, asset reserves can facilitate rural residents’ participation in commercial pension insurance through diverse pathways.

Models (2) to (5) demonstrate that the gender variable is statistically significant at the 10% level concerning individual characteristic control factors, suggesting that rural male inhabitants are less inclined to acquire commercial pension insurance than their rural female counterparts. In rural China, men typically act as the primary earners for their families, shouldering more financial burdens and social obligations. Consequently, male inhabitants must prioritize their immediate survival requirements and familial financial obligations, relegating pension preparation to a secondary status. Secondly, rural men and women hold distinct beliefs and varying levels of acceptability regarding risk. Male residents generally exhibit a greater propensity for risk and have a heightened capacity to endure danger, operating under the belief that they may address the pension issue in the future through alternative strategies. Women exhibit heightened sensitivity to risk and demonstrate a propensity to acquire insurance to mitigate the uncertainties associated with their retirement. The resident’s age is significantly more likely to influence participation in commercial pension insurance at the 1% statistical level, whereas the “age^2^/100” of the resident exerts a significant adverse effect on participation in commercial pension insurance. The influence of age on enrollment in commercial pension insurance exhibits a non-linear relationship, characterized by an inverted “U”-shaped curve that ascends and subsequently descends, aligning with the findings of the existing literature (57). As rural inhabitants age, their cognizance of eldercare intensifies, prompting them to consider future old-age support issues. At this juncture, the probability of engaging in commercial pension insurance increases; however, once a resident surpasses a specific age threshold, the availability of suitable insurance products diminishes. The requisite annual premium escalates, reducing the inclination to participate in insurance. The educational attainment of rural dwellers substantially enhances the likelihood of engaging in commercial pension insurance. As the educational attainment of rural dwellers increases, so does their understanding of commercial pension insurance as a tool for future risk management, which can help preserve their quality of life in old age. Simultaneously, highly educated individuals possess broader avenues for acquiring information. They can leverage their extensive experience and information to make informed decisions on acquiring commercial pension insurance by analyzing the insurance market dynamics and policy modifications. The trait of “holding financial assets” significantly positively influences participation in commercial pension insurance. The situation arises from their prior understanding of the financial market, gained through earlier activities, which has led to an awareness of the uncertainty surrounding returns on financial assets. The distinction lies in that business pension insurance, as a specialized financial instrument, entails comparatively low risks and offers predictable profits and long-term security, thereby explaining their greater willingness to engage in commercial pension insurance. Nonetheless, the marital status, health, family size, and risk appetite of rural inhabitants do not exert a substantial influence on their engagement in commercial pension insurance.

### 5.2 Robustness examination

This research conducts a robustness test to assess the trustworthiness of the regression results regarding children’s cohabitation and household asset reserves on commercial pension insurance, with the specific results presented in Table 4.

**Table 4:** Robustness test.

Variable	Model (1)	Model (2)
	Random sample	OLS
Living	−0.452**	−0.043**
	(0.190)	(0.017)
Cash	0.047**	0.004***
	(0.023)	(0.001)
House	0.299***	0.055^***^
	(0.105)	(0.018)
Car	0.520***	0.064***
	(0.147)	(0.017)
Control	YES	YES
PseudoR^2^	0.4455	–
*R* ^2^	–	0.3264
*N*	1,000	1,386

Robust standard errors in parentheses ***, **, and * indicate significance at the 1, 5, and 10% levels, respectively.

#### 5.2.1 Modify the sample capacity

To mitigate the potential for regression results influenced by sample selection bias during data collation, this study employed random sampling without replacement from the original 1,586 valid samples, established 50 random seeds, and ultimately extracted 1,000 subsamples. The probit model was subsequently employed for regression analysis, and the yielding model was used (1).

#### 5.2.2 Model substitution

The Probit model ensures the validity of the probability interpretation and accommodates the non-linear properties of the error term when the explanatory variables are dichotomous. However, it has limits in interpreting linear relationships. The OLS model is a linear regression technique appropriate for continuous dependent variables, yielding brief and easily interpretable results. In this study, the Probit model is substituted with the OLS model for regression to obtain model (2). The comparison of Tables 3, 4 reveals that the results are fundamentally similar, except for variations in coefficients and standard errors. The data indicates that cohabitation among children markedly diminishes the probability of rural residents engaging in commercial old-age insurance. In contrast, asset reserves substantially enhance this likelihood, corroborating hypotheses H1 and H2.

### 5.3 Endogeneity test

This paper employs the instrumental variable method to evaluate endogeneity and prevent endogeneity issues that may arise from omitted variables or reciprocal causality in the model regression. Firstly, this article uses “economic independence” as an instrumental variable for children cohabiting in the same household, referred to as “Independence” in the text. The economic independence of parents and children directly influences the decision of whether children reside with their parents, as seen in the correlation. Older parents may encounter financial instability due to limited career options, or their children may remain financially dependent on them. In the aforementioned scenario, the likelihood of children cohabiting with their parents will markedly rise, particularly in rural regions. From an exogenous perspective, economic independence itself does not directly influence parents’ decisions to purchase insurance. Parents’ perspectives regarding geriatric care, risk awareness, and life cycle planning are the primary factors that influence the demand for pension insurance. At the same time, economic independence is not related to these factors. Secondly, this paper employs factor analysis to consider “cash,” “house,” and “car” as variables in the “Factor” variable. Then, it employs the “household income” variable as the instrumental variable for the “Factor” variable, as household asset reserves are divided into three aspects. From the perspective of correlation, household income, as the primary source of household asset reserves, directly determines the household’s savings capacity and asset allocation structure. From an exogenous perspective, household income levels do not directly influence commercial pension insurance purchase decisions; their impact is transmitted only through asset reserves. Similarly, the demand for insurance is influenced by factors such as attitudes toward aging, risk perception, and life cycle planning. In contrast, household income, an exogenous economic variable, serves as an independent error term in the decision to participate in insurance. Although income levels may indirectly influence insurance participation capacity through consumption patterns, their direct impact can be isolated by regulating variables such as household debt and family structure.

According to Table 5, the *F*-value of the weak instrumental variable test rejects the original hypothesis. This paper employs a two-stage least squares regression analysis, revealing that, after addressing the endogeneity issue through the instrumental variable approach, cohabiting children significantly deter residents’ engagement in commercial pension insurance, while family asset reserves markedly enhance residents’ participation in commercial pension insurance.

**Table 5:** Endogeneity test.

Variable	Model (1)	Model (2)	Model (3)	Model (4)
	Living	Insurance	Factor	Insurance
Living		−0.061***		
		(0.017)		
Independence	0.973***			
	(0.008)			
Factor				0.729***
				(0.205)
Income			0.119***	
			(0.033)	
Control	YES	YES		
*F*	23.43		94.85	
*N*	1,386	1,386	1,386	1,386

Robust standard errors in parentheses ***, **, and * indicate significance at the 1, 5, and 10% levels, respectively.

### 5.4 Analysis of interaction effects

The benchmark regression model indicates that cohabitation with children significantly inhibits rural residents’ participation in commercial pension insurance. At the same time, family asset reserves significantly promote participation in commercial pension insurance, with results remaining stable through robustness testing. Is there a correlation between cohabiting children and family asset reserves? What is the impact of the interaction? Investigating this subject is equally beneficial for the advancement of commercial pension insurance. When fathers and children cohabit, intergenerational labor assistance and reciprocal caregiving enhance fathers’ quality of life in later years. However, with limited family asset reserves, the crowding-out effect on engagement in commercial pension insurance may be more significant. Conversely, when children and parents are not cohabiting, fathers may independently address the challenge of financial resources in later life. However, families with more significant asset reserves exhibit a heightened sense of precautionary savings and demonstrate increased sensitivity to pension asset reserves, making them more inclined to acquire commercial pension insurance. Consequently, an interaction mechanism may exist between cohabiting children and household asset reserves. This study initially focuses on the two primary explanatory variables: children’s presence and asset reserves. It subsequently creates three distinct interaction terms and incorporates these terms into the prior analysis for regression analysis. The outcomes will be presented in Table 6.

**Table 6:** Analysis of interaction effects.

Variable	Model (1)
c_living	−1.086***
	(0.268)
c_crash	0.077***
	(0.021)
c_house	0.298**
	(0.131)
c_car	0.596***
	(0.158)
c_living×c_crash	0.128***
	(0.048)
c_living×c_house	0.672**
	(0.274)
c_living×c_car	0.592*
	(0.367)
Control	YES
*N*	1,386
PseudoR^2^	0.4647

Robust standard errors in parentheses ***, **, and * indicate significance at the 1, 5, and 10% levels, respectively.

Table 6 illustrates that while the coefficient representing the impact of children’s cohabitation on participation in commercial pension insurance is negative, the coefficients of the three interaction terms regarding rural residents’ participation in commercial pension insurance are positive and exhibit a significant enhancing effect. This phenomenon indicates that family asset reserves substantially mitigate the adverse impact of children’s cohabitation on rural residents’ engagement in commercial pension insurance. The primary reason children cohabiting impedes engagement in commercial pension insurance is that parents believe they can entirely depend on their offspring for essential financial stability in their later years. Nevertheless, for individuals possessing substantial family asset reserves, parents begin to reconsider the matter of aging. They recognize that while their offspring may assist them in their later years, the unpredictability of their future circumstances could impose more financial burdens on their children, ultimately jeopardizing the quality of life for their parents in old age. Consequently, dads are likely to engage in commercial pension insurance to secure financial autonomy in their later years, reduce their children’s financial obligations, and simultaneously relish the companionship of their offspring. Consequently, the asset reserve has diminished the restraining influence of children’s cohabitation on engagement in commercial old-age insurance.

Given the potential for a non-linear relationship between the core explanatory factors and the explanatory variables, the regression coefficients of the interaction terms in the Probit model may be spuriously significant. This paper examines the changes in the marginal effects of children’s cohabitation and family asset holdings on participation in commercial pension insurance, using graphs to analyze the significance, magnitude, and direction of the interaction term’s effect.

**Figure 3:**
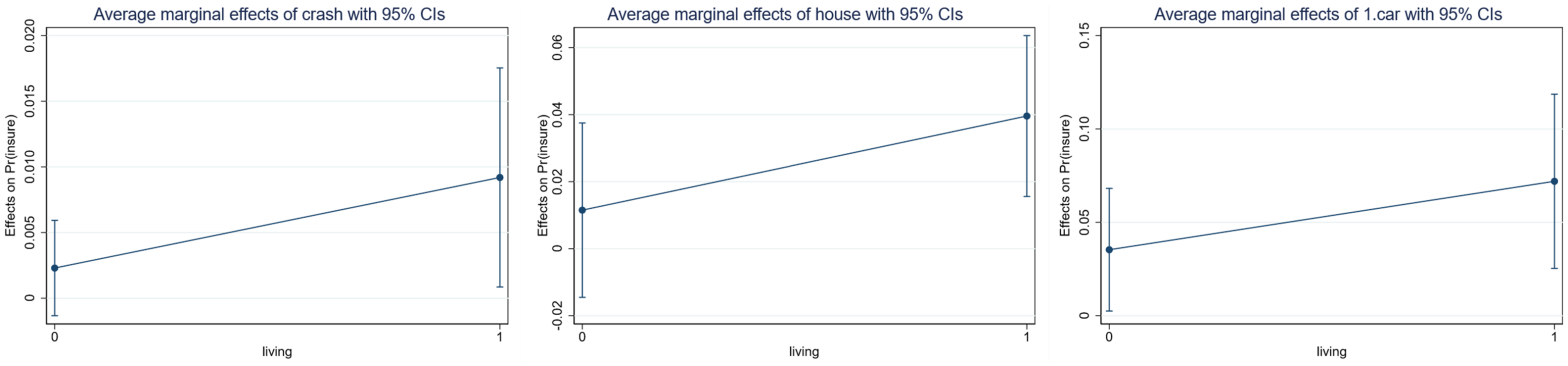
The marginal effect of household asset reserves on participation in commercial pension insurance when children live at home.

The horizontal axes of Figure 3 represent children’s cohabitation. In contrast, the vertical axes depict the average marginal effects of cash stock, property count, and automobile ownership on rural residents’ engagement in commercial pension insurance. The graph indicates that asset stock has a positive influence on the likelihood of participating in commercial pension insurance, regardless of the father’s cohabitation with his children, with marginal effects increasing progressively.

**Figure 4:**
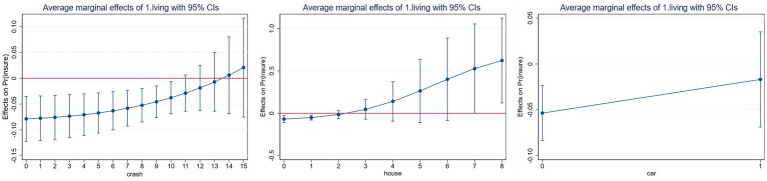
The marginal effect of having children living at home on participation in commercial pension insurance in the case of family asset reserves.

The horizontal axis of Figure 4 represents cash stock, the quantity of properties, and the automobile status, while the vertical axis illustrates the average marginal effect of children’s cohabitation on rural residents’ engagement in commercial pension insurance. The figure demonstrates that as cash reserves and property ownership increase, the marginal impact of children’s homeliness on participation in commercial pension insurance progressively shifts from negative to positive. Furthermore, while the marginal effect of automobiles on participation in commercial pension insurance remains negative, its magnitude diminishes significantly, indicating that automobile ownership substantially mitigates the inhibitory impact of children’s homeliness on participation in commercial pension insurance.

The probability of rural residents participating in commercial pension insurance is significantly increased by the moderating effect of family asset reserves and children’s cohabitation, as demonstrated in Figures 3, 4. Additionally, family asset reserves significantly weaken the inhibitory effect of children’s cohabitation on commercial pension insurance. Initially, asset reserves undermined conventional support obligations by altering intergenerational authority dynamics. As family assets increase, the enhanced economic autonomy of the older adult cohort facilitates the reclamation of decision-making authority, disrupting the intergenerational transfer of the children’s “veto power to insure” and elevating the likelihood of insurance participation. Secondly, the accumulation of assets correlates with an enhancement in financial literacy, encouraging households to reevaluate their understanding of risk. Residents are increasingly recognizing that commercial pension insurance is a crucial component of asset allocation, as it mitigates risks and enhances the value of assets. Ultimately, Children’s Cohabitation and old-age insurance may constitute a complementing synergy. Rural inhabitants dismiss old-age insurance due to the lack of spiritual solace from their offspring. However, the reliable financial assistance of commercial old-age insurance alleviates the economic strain of their children’s later years. Consequently, children may integrate both factors in their retirement decision-making, providing parents with emotional value while alleviating their children’s financial burden.

### 5.5 Analysis of heterogeneity

China is an expansive nation characterized by significant disparities in resource allocation and familial pension practices across its rural regions. For instance, the cognitive capabilities of rural inhabitants vary according to their educational attainment. At the same time, differences in health status influence attitudes toward pension options and the aging process. The abovementioned situations alter the likelihood of rural residents engaging in commercial pension insurance. Consequently, this paper will examine the heterogeneity across two dimensions: educational attainment and physical health status, which is highly significant for developing commercial pension insurance in China. The findings are presented in Table 7.

**Table 7:** Heterogeneity analysis.

Variable	Model(1)	Model(2)	Model(3)	Model(4)
	Primary school and below	Above primary school	Unhealthy	Health
Living	-0.869**	-0.176	1.663	-0.543***
	(0.430)	(0.233)	(1.645)	(0.189)
Wald	0.8672		0.0247	
Cash	-0.010	0.073**	-0.137	0.045**
	(0.026)	(0.029)	(0.087)	(0.020)
Wald	0.0017		0.4984	
House	0.135	0.351***	0.704	0.331***
	(0.242)	(0.116)	(0.846)	(0.101)
Wald	0.0405		0.0111	
Car	0.599**	0.305*	1.759**	0.410***
	(0.246)	(0.179)	(0.730)	(0.143)
Wald	0.4341		0.2179	
Control	YES		YES	
PseudoR^2^	0.1197	0.5280	0.4636	0.4548
*N*	851	532	234	1150

Robust standard errors in parentheses ***, **, and * indicate significance at the 1, 5, and 10% levels, respectively.

#### 5.5.1 Education heterogeneity

The participation of inhabitants in the low-education group in commercial pension insurance is significantly inhibited by the cohabitation of children, as evidenced by models (1) and (2). The crowding-out effect on commercial pension insurance participation is exacerbated by the conventional pension models that rural residents adhere to, such as children’s responsibility, due to their shorter years of education and lagging pension concepts. There is no significant inhibitory effect on highly educated residents, as they are more likely to adopt commercial pension insurance.

Among the asset reserves, cash holdings and property ownership substantially influence the engagement of highly educated individuals in commercial pension insurance. In contrast, car ownership has a relatively insignificant impact on highly educated rural inhabitants. Overall, asset reserves are crucial in engaging highly educated individuals in commercial pension insurance. On the one hand, highly educated individuals in rural regions typically possess superior job advancement prospects and a more stable economic foundation, as evidenced by their family’s asset level. Residents possessing more significant household asset reserves will exhibit enhanced purchasing power, enabling them to allocate more resources toward commercial pension insurance participation. On the other hand, highly educated individuals possess superior information comprehension and processing abilities, making them more adept at understanding details regarding premiums, coverage, and risks associated with commercial pension insurance. This proficiency significantly bolsters trust in insurance participation and elevates the probability of engaging in commercial pension insurance.

#### 5.5.2 Health heterogeneity

According to models (3) and (4), the *p*-value for the between-group difference in health degrees is 0.0247, indicating a statistically significant disparity in health degrees. Further, it suggests that the coefficient of children’s cohabitation on the participation of healthy residents in commercial old-age insurance is more significant and exhibits a more pronounced inhibitory effect. Healthy individuals often underestimate their future health concerns, equating current health with future well-being, which results in a diminished sense of urgency as they age. Overall, household asset reserves substantially enhance the engagement of healthy residents in commercial pension insurance, whereas ill resident groups exhibit reduced sensitivity to this factor. Unhealthy population groups incur additional medical expenses, which diminishes their financial resources for future retirement planning, reducing their inclination toward commercial pension insurance. Conversely, healthier individuals have a greater life expectancy and a more optimistic outlook toward retirement, enhancing their propensity to engage in commercial pension insurance.

## 6 Conclusions and implications

This study examines the impact of children’s cohabitation and household asset holdings on rural residents’ participation in commercial pension insurance, utilizing the CFPS 2018 and 2020 databases, and presents the resulting conclusions.

(1) The benchmark regression model revealed that children’s cohabitation significantly reduces the likelihood of rural residents participating in commercial old-age insurance. In contrast, household asset reserves substantially enhance participation, thereby preliminarily validating hypotheses H1 and H2. In order to verify the reliability of the conclusion, this paper uses three methods to carry out the robustness test. At the same time, to avoid the bias caused by endogeneity, this paper uses the instrumental variable method to conduct endogeneity tests, and its regression results are consistent with the previous ones, preliminarily verifying H1 and H2.
(2) This study analyzes the impact of interaction terms between children’s cohabitation and family asset reserves on participation in commercial pension insurance. The regression analysis reveals that all three interaction terms significantly enhance the likelihood of rural residents engaging in commercial old-age insurance. This result suggests that family asset reserves mitigate the negative impact of children’s cohabitation on participation in commercial old-age insurance. Furthermore, the positive influence of family asset reserves on insurance participation is notably more substantial than the negative influence of children’s cohabitation.
(3) The results from the heterogeneity analysis indicate that children’s cohabitation has a dampening effect on the participation of residents with low education and health in commercial pension insurance. However, household asset reserves have a significant influence on participation in commercial pension insurance among highly educated residents and those in good health.

This paper presents the following recommendations:

Initially, raise awareness of old-age care and change people’s attitudes. The state should enhance the development of the pension legal framework, promote village collective-based pension mutual aid organizations, and encourage older adult participation in pension insurance through neighbor mutual aid models, thereby increasing confidence in commercial pension insurance participation. The government should enhance the regular dissemination of information regarding the protection of the rights and interests of the older adult. Such activity should involve the integration of typical cases, the reinforcement of the legal obligations associated with older adult support, and the clarification of these obligations along with their legal ramifications, thereby fostering a greater sense of responsibility for family support. Village cadres should organize clear and accessible lectures on pension insurance, incorporating practical examples of the benefits of participation. This approach aims to dispel the misconception among rural residents that “participation in the insurance is useless” and enhance their understanding of self-supporting pensions.

Additionally, enhancing farmers’ income while lowering insurance expenses. The absence of labor markets and employment opportunities in rural regions, combined with inadequate family asset reserves, has diminished economic independence in old age, necessitating reliance on children for support during this period. Local governments should develop rural specialty industries tailored to local conditions, accelerate rural infrastructure development, and enhance employment skills training and re-education programs to support rural communities. These measures aim to increase local employment opportunities, improve the quality of life for rural residents, and broaden their income sources, ultimately achieving more stable incomes for farmers. Secondly, the Government should implement policies incentivizing insurance organizations to create preferential commercial pension insurance products for farmers. Additionally, tax incentives and subsidies would reduce the cost barrier for rural residents to use commercial pension insurance.

Eventually, enhancing insurance products and assist potential users. Initially, local governments should conduct regular insurance awareness campaigns for residents with low levels of education, inviting insurance company professionals to provide in-person explanations and education. Additionally, they may employ social media and brief videos to elucidate the fundamental concepts of commercial pension insurance. Insurance institutions must help rural residents understand the importance and necessity of commercial pension insurance, while also building their confidence in these products. Simultaneously, insurance institutions should refrain from employing abstruse and hard-to-understand professional terminology when developing claims settlement guidelines. Instead, they should employ more relatable examples and colloquial expressions. Collective insurance enrollment models and agency enrollment models should be actively promoted through village committees. Then, insurance institutions should expand the age range of pension insurance coverage by creating more customized commercial pension insurance products specifically designed for individuals approaching retirement. The government should promote early retirement planning for younger residents and the purchase of commercial pension insurance products in advance. Lastly, the scope of coverage and claim settlement conditions should be modified to accommodate residents with poor health conditions. For instance, the flexibility of claim settlement should be improved, and coverage for specific diseases should be included. Furthermore, Government agencies should mandate that insurance providers streamline the enrollment process and minimize the time investment required for residents to understand the program, thereby facilitating prompt enrollment.

## Data Availability

The raw data supporting the conclusions of this article will be made available by the authors, without undue reservation.
